# Metabolic abnormalities in adult HIV infected population on antiretroviral medication in Malaysia: a cross-sectional survey

**DOI:** 10.1186/1471-2458-13-758

**Published:** 2013-08-15

**Authors:** Nazisa Hejazi, Roslee Rajikan, Christopher Lee Kwok Choong, Suzana Sahar

**Affiliations:** 1 Dietetics Program, School of Health Sciences, Faculty of Health Sciences, Universiti Kebangsaan Malaysia, Jalan Raja Muda Abdul Aziz, Kuala Lumpur, 50300, Malaysia; 2 Jabatan Perubatan Am, Hospital Sungai Buloh, Jalan Hospital Sungai Buloh, Selangor, Darul Ehsan, 47000, Malaysia

**Keywords:** Lipid disorders, Metabolic abnormalities, Fasting plasma glucose, ARV medication, Protease inhibitor, Cardiovascular disease, Dyslipidemia, HIV positive, Malaysia

## Abstract

**Background:**

In the current two decades, dyslipidemia and increased blood glucose as metabolic abnormalities are the most common health threats with a high incidence among HIV/AIDS patients on antiretroviral (ARV) treatment. Scientific investigations and reports on lipid and glucose disorders among HIV infected communities are inadequate especially in those developing such as Malaysia. This cross-sectional survey was mainly aimed to evaluate the prevalence of metabolic abnormalities and associated risk factors among HIV infected population patients on ARV medication.

**Methods:**

In a single reference health center in Malaysia, 2739 adult HIV positive patients on antiretroviral therapy (ART) were studied cross-sectionally using medical records. Besides demographic variables and associated health disorders, those factors which can change the lipid and glucose levels were collected. Logistic Regression was used to find the potential risk factors (p < 0.05).

**Results:**

Majority of the studied population were male (81.1%) and aged between 30–49 (68.6%). Mean CD4 count was 474.25 (cells/mm^3^) while undetectable RNA viral load was common among 83.3 (%) of subjects. Among 1,583 patients with the recent blood lipid and glucose tests, increased levels of triglyceride (TG) and total cholesterol (TC) were frequently prevalent in half of the population as 59 (%) and 54.2 (%) while 28.7 (%), 35.1 (%) and 38.2 (%) had declined level of high-density lipoprotein (HDL), raised low-density lipoprotein (LDL) and fasting plasma glucose (FPG) which were less common. Dyslipidemia was common in 82.3 (%) of the subjects. Notably, medication with protease inhibitor (PI) was a potential risk for elevated triglyceride (odds ratio (OR) = 2.309, 95% confidence interval (CI) = 1.605–3.324, P = 0.001), high TC (OR = 1.561, 95% CI = 1.123–2.169, P = 0.008) and low HDL (OR = 1.449, 95% CI = 1.037–2.024, P = 0.029). As lifestyle factor, alcohol consumption results as significant risk factor for raised TG (OR = 2.653, 95% CI = 1.353–5.202, P = 0.004). Also having hepatitis raised risk of high FPG level (OR = 1.630, 95% CI = 1.197-2.220, P = 0.002) in this sample population.

**Conclusions:**

Dyslipidemia is highly common in Malaysian HIV subjects receiving ARV medication. Lifestyle modification, changing PI and switch to other ARV regimen can help in reduction of these abnormalities. Also suitable strategies and plans are necessary to prevent cardiovascular diseases in future.

## Background

Human immunodeficiency virus infection/acquired immunodeficiency syndrome (HIV/AIDS) is still considered as a tragic infectious disease with a high impact on quality of life among individuals and communities through the weakening economic and social performances which manifests along with poor mental and/or physical health. Based on the latest report by The Joint United Nations Programme on HIV/AIDS (UNAIDS), of 34.0 million [31.4 million- 35.9 million] people living with HIV (PLHIV) at the end 2011 universally [1]. Roughly 81,000 of them [2] were resident in Malaysia which is a south-east Asian and Upper middle-income country [3]. Indeed the number of PLHIVs receiving ART as of December 2011 has increased up to 14,002 [2]. In combat with HIV/AIDS, there are some progresses in heath condition, quality of life and life expectancy specific after introduction of antiretroviral therapy (ART) as the only available medication for control of this disease [4]. However there has been discovered some adverse effects of ART in the form of glucose and/or lipid abnormalities which are accounted as cardiovascular risk factors [5]. The prevalence rates for these disorders are widely varied ranging from less than 30% for elevated glucose level [6-9] and 10%-20% for diabetes [10-13], 10-60% for hypercholesterolemia [14-17], 20-70% for hypertriglyceridemia [15,18,19], 20-40% for low HDL level [18-20], which are mostly in the form of elevated triglyceride and low HDL level. In reviews by Carr et al. (1998), Carr (2000) and Currier et al. (2008) on substantial HIV/AIDS studies they explained other than traditional risk factors and HIV infection itself [21-23]. They stressed that protease inhibitor (PI) as the third antiretroviral (ARV) class also is responsible for these complications in comparison with other ARV categories such as nucleoside analog reverse transcriptase inhibitors (NRTI) and non-nucleoside analog reverse transcriptase inhibitors (NNRTI) and integrase inhibitors (INI).

Generally, the knowledge on blood metabolic abnormalities among PLHIV have established mostly in developed societies including USA [24-26], Europe [27,28], Australia [29-32] and limited information in developing [16,19,33,34] and African countries [35-37].

In Asian and specific South east Asian countries estimated incidence of lipid abnormalities in HIV/AIDS mostly are reported from Thailand [15,33,38-40] and India [7,41,42]. They found a very wide range of 5-60%for high TC, high LDL-c, high TG and low HDL among antiretroviral therapy (ART) individuals. Elevated FPG also is estimated between estimated 27% among Thai [6,38] and 60% in Indian HIV population [7].

The aim of this study is to provide some evidences on metabolic abnormalities and also associated risk factors since there is very limited information from HIV/AIDS studies about these disorders in Malaysia. Moreover, information from the developed countries cannot be deduced to developing societies due to differences in patient populations, HIV subtypes and lifestyle therefore, therefore initial and additional studies from developing countries are required. we assessed the prevalence of lipid and glucose abnormalities and also associated the risk factors with each these abnormalities among Malaysian HIV-infected patients in this study.

## Methods

### Study population and design

This cross-sectional study was conducted between January to September 2012 at an Infectious Diseases (ID) clinic which is also a reference center for HIV and AIDS cases, located in Selangor, Malaysia. All HIV patients who started their ARV medication till first January 2012 were listed based on the computerized medical records. Patients included in this study met the following criteria: subjects must have been on antiretroviral medication at least for 6 months and stable HAART for at least 4 weeks before study entry, had complete or at least partial lipid and glucose panel performed during this study, equal or more than 20 years of age and hold Malaysian citizenship. All death cases were excluded.

Ethical approval was obtained from research ethic committee/IRB National University of Malaysia and National Medical Research Registry (NMRR) Malaysia.

### Methods

The collected secondary data from computerized medical records comprised of demographics (age, gender, and ethnicity), weight, height and blood pressure, multivitamin supplementation, ARV medication (NRTIS, NNRTIS, PIs), those medication history which have effect on lipid and or glucose level (anti-hypertensive, diuretic, steroid/hormone, lipid-lowering, anti-hyperglycemic). Also information regarding severe opportunistic infections during preceding six months, medical history as the major current health problem (tuberculosis, diabetes mellitus, hepatitis, other liver and renal diseases), alcohol and smoking status including the number of subject as current alcohol consumer, current smoker, and the number of smoked cigarettes per day were collected.

Fasting blood samples were collected for all studied subjects. Laboratory testing for blood samples included the following: fasting lipid profile, fasting plasma glucose, CD4 count, RNA viral load. The laboratory methods for determination of lipid profiles includes Cholesterol Oxidase-Peroxidase Amino Phenazone Phenol (CHOD-PAP) for TC, Accelerator Selective Detergent for HDL,Glycerol-3-Phosphate Oxidase-Peroxidase Amino Phenazone Phenol (GPO-PAP) for TG while Low-density lipoprotein (LDL) was derived from an indirect measurement using the Friedewald formula [43] . Blood TC, TG and HDL cholesterol was tested using the Cobas Integra 400 Plus analyzer (Roche Diagnostics Ltd. CH6343, Rotkreuz, Switzerland). Blood glucose was tested by CD4 was measured using the FACS Calibur system (Becton Dickinson, San Jose, CA). HIV RNA Viral load was detected with Immunophenotyping method using COBAS®AMPLICOR analyser.

### Study definitions

Lipid abnormalities were defined according tothe National Cholesterol Education Program (NCEP) Adult Treatment Panel III (ATP III) criteria in 2001 [44] while the American Heart Association (AHA) and the National Heart, Lung, and Blood Institute (NHLBI)/Updated NCEP [45,46] criteria was applied for plasma glucose level. Abnormalities in lipid profile and glucose level (mmol/l) were classed as TG ≥ 1.7, LDL ≥ 3.36, TC ≥ 5.17, HDL < 1.03 (male) and HDL < 1.30 (female), FPG ≥ 5.6. The definition of dyslipidemia was according to the occurrence of one or more of the lipid abnormalities (TG, TC, LDL, HDL) according to the criteria of the National Cholesterol Education Program Adult Treatment Panel III.

The CD4 cell counts (cells/mm^3^) were categorized in there groups (< 200, 200–499, ≥ 500) according to the standard of Centre for Disease Control and Prevention [47]. Also HIV RNA load (copies/mL) was classified using the recommendation from International AIDS Society-USA Expert Panel [48] references into undetectable level (value below 20) and detectable level (as equal or greater than 20).

### Statistical

#### Analysis

All collected data were analyzed using the statistical packages for social science (SPSS) for window, version 16.0 [49]. Descriptive data in the form of mean ± standard deviation (SD) and frequency as the number and percent (%) were tested. At the beginning, all variables were tested for normality distribution and then data cleaning was carried out to weed out the out-of-range numbers. At the second step of data analysis, based on the lipid and glucose data (normal and non-normal groups) the means and percentage distribution of each variable were calculated using independent sample-t-test and chi-square test of homogeneity to find significant differences and associations in variables. As the last step, the significant risk factors for each metabolic abnormality using adjusted odd ratios, 95% confidence intervals and p-values were identified through univariate test as binary logistic regression with enter method. All binary analyses were adjusted for age, gender, ethnicity, hypertension, diabetes mellitus, CD4, HIV RNA Load, TC, TG, LDL, HDL, FPG, alcohol consumption, smoking, liver disease, medication with agents including ARV (PI), b-blocker, anti-hyperglycemic. The significance tests were 2-sided, and P values less than 0.05 were considered statistically significant.

## Results

### Characteristics of study population

Two thousand seven hundred thirty nine patients were included in the analysis. Dyslipidemia was common in 82.3% of 1583 HIV subjects. Demographics are shown in Table 1. Most subjects were aged between 40 to 59 years (55.6%), Chinese (58.8%) and male (81.1%). Mean systolic (121.31 ± 16.84 mmHg) and diastolic blood pressure (76.83 ± 11.40 mmHg) were in normal range while 19.7% of 2670 subjects were hypertensive. In terms of ARV medication of all 2637 subjects on ARV treatment 9.3% was receiving PIs and the rest were on the combination of NRTIs + NNRTIs medication. Small proportions of subjects were taking anti-hypertension agents (12.2%) and lipid lowering (18.5%). Majority of HIV study population (89.2%) had CD4 cell count ≥ 200 (cells/mm^3^) and also 83.3% had a well suppressed and undetectable HIV viral load as less than 20 (copies/mm^3^). In relation to lipid abnormalities, a smaller group of subjects (26.7%) had the lipid test result only for year 2011 in the time of data collection. The lipid profile results from year 2012 showed that increased level of triglyceride (> 1.7 mol/ml) was common among 59.1% of subjects while 35.1% and 54.2% of subjects had higher amount of LDL and total cholesterol. HDL was lower than normal level in 28.7% of subjects and 38.2% had high fasting plasma glucose level.

**Table 1:** Characteristics of 2739 HIV infected Subjects on ARV medications

**Characteristics**	**N (%)**	**Mean ± SD**
**Age**		43.06 ± 9.90
20-39	1053 (38.5)	33.56 ± 4.35
40-59	1524 (55.6)	47.22 ± 5.14
≥ 60	162 (5.9)	65.70 ± 4.82
**Ethnicity**		
Malay	836 (30.5)	
Chinese	1611 (58.8)	
Indian	274 (10.0)	
Other ^¦^	18 (0.7)	
**Gender**		
Male	2220 (81.1)	
Female	519 (18.9)	
**Weight (Kg)**	2582 (94.27)	61.85 ± 1.21
**Height (cm)**	215 (7.85)	165.90 ± 9.62
Hypertension*	514 (19.7)	
**ARV without PI exposure**	2392 (90.7)	
**ARV with PI exposure**	245 (9.3)	
**Supplement**^**§**^	384 (14)	
**B-blocker**	70 (2.6)	
**Other than B-blockerª**	263 (9.6)	
**Diuretic**	15 (0.5)	
**Steroid/Hormone**	6 (0.2)	
**Lipid-lowering agents**	508 (18.5)	
**Anti-Hyperglycaemic agent**	191 (7.0)	
**CD4 (cells/mm**^**3**^**)**	2739 (100.0)	474.25 ± 263.02
**CD4 < 200 (cells/mm**^**3**^**)**	292 (10.8)	122.94 ± 53.78
**HIV RNA Load < 20 (copies/mL)**	2282 (83.3)	
**TG ≥ 1.7 (mmol/l)**	933(59.1)	3.27 ± 1.92
**LDL-C ≥ 3.36 (mmol/l)**	555 (35.1)	4.10 ± 0.75
**TC ≥ 5.17(mmol/l)**	857 (54.2)	6.16 ± 0.95
**Low HDL (mmol/l)**^**b**^	454 (28.7)	
**FPG ≥ 5.6 (mmol/l)**	588 (38.2)	7.08 ± 2.24
**Dyslipidaemia**	1303 (82.3)	
**Current alcohol consumer**	83 (3.0)	
**Current smoker**	288 (10.5)	
**Diabetes mellitus**	352 (12.9)	
**Tuberculosis**	46 (1.7)	
**Hepatitis**	395 (14.4)	
**Renal diseases**	51 (1.9)	

¦ Sarawakian and Sabahan *Blood Pressure ≥ 140/90 (mmHg). §Multivitamin and vitamin (B complex and C). ª Calcium channel blocker, Alpha blockers (ACE inhibitors, Angiotensin II receptor antagonists). *SD* Standard deviation. ^b^HDL (mmol/l) < 1.03 in male, < 1.30 in female.

Also in year 2012 till September 10.5% of study population were current smoker that 46.5% of smokers reported more than 20 smoked cigarettes per day. Prevalence of diabetes mellitus and Hepatitis are estimated as 12.9% and 14.4% as the most common medical associated condition among this population. HDL (Mean + SD) level in male (0.89 + 0.12 mmol/l) was lower than and female (1.08 + 0.17 mmol/l) groups.

### Risk factors of high TG

The result of logistic regression analysis (Table 2) revealed that significant risk factors (p < 0.001) for increased TG level were increasing age (OR = 1.018, 95% CI = 1.008 -1.029), having hypertension (OR = 1.516, 95% CI = 1.173 - 1.960) and diabetes mellitus (OR = 1.532, 95% CI = 1.150- 2.040), taking b-blockers as antihypertensive agents (OR = 1.668, 95% CI = −1.042), higher FPG (OR = 1.166, 95% CI = 1.084-1.253), higher CD4 cell count (OR = 1.001, 95% CI = 1.001-1.002), higher level of TC (OR = 1.281, 95% CI = 1.179 - 1.392) with following strong risks as low HDL level (OR = 3.585, 95% CI = 2.779-4.625), alcohol taking (OR = 2.653, 95% CI = 1.353- 5.202) and ARV therapy with PIs (OR = 2.309, 95% CI = 1.605- 3.324).

**Table 2:** Risk factors for increased triglyceride (TG) in 1579 HIV subjects (normal = 646, increased TG = 933) on ARV medication

**Characteristics**	**Normal**	**Increased triglyceride**	**aOR**	**95.0 C.I. for OR**		**p-value**
	**Mean ± S.D.**	**Mean ± S.D.**		**Lower**	**Upper**	
	**N (%)**	**N (%)**				
**Age (per year)**	43.41 ± 10.51	45.14 ± 9.36	1.018	1.008	1.029	0.001
**Gender**						
Male	484 (74.9)	788 ( 84.5)	1.00			
Female	162 (25.1)	145 ( 15.5)	0.550	0.428	0.707	0.001
**Ethnicity**						
Indian	62 (9.7)	96 (10.3)	1.00			
Malay	190 (29.8)	199 (21.4)	0.676	0.464	0.985	0.042
Chinese	386 (60.5)	635 (68.3)	1.062	0.754	1.498	0.730
**Hypertension**						
No	515 (82.4)	670 (75.5)	1.00			
Yes	110 (17.6)	217 (24.5)	1.516	1.173	1.960	0.010
**ARV medication**						
Without PI	591 (93.6)	786 (85.9)	1.00			
With PI	42 (6.4)	129 (14.1)	2.309	1.605	3.324	0.001
**b-Blocker**						
Not taking	620 (96.0)	872 (93.5)				
Taking	26 (4.0)	61 (6.5)	1.668	1.042	2.670	0.033
**Anti-hyperglycaemic agent**						
Taking	45 (7.0)	103 (11.0)				
Not taking	601 (93.0)	830 (89.0)	0.603	0.419	0.870	0.006
**Diabetes mellitus**						
No	565 (77.5)	765 (82.0)	1.00			
Yes	81 (12.5)	186 (18.0)	1.532	1.150	2.040	0.004
**TC (mmol/l)**	5.05 ± 1.00	5.43 ± 1.40	1.281	1.179	1.392	0.001
<5.17	363 (56.2)	361 (38.7)	1.00			
≥5.17	283 (59.1)	571 (40.9)	2.029	1.655	2.487	0.001
**HDL (mmol/l)**	1.43 ± 0.44	1.24 ± 0.61	0.485	0.384	0.612	0.001
Normal^§^	551 (85.3)	576 (61.8)	1.00			
Low	95 (14.7)	356 (38.2)	3.585	2.779	4.625	0.001
**FPG (mmol/l)**	5.52 ± 1.37	5.91 ± 1.95	1.166	1.084	1.253	0.001
Normal	432 (68.7)	519 (57.2)	1.00			
High	197 (31.3)	389 (42.8)	1.644	1.327	2.035	0.001
**Alcohol**						
Not Taking	635 (98.3)	892 (95.6)	1.00			
Taking	11 (1.7)	41 (4.4)	2.653	1.353	5.202	0.004
**Hepatitis**						
No	546 (84.5)	844 (90.5)				
Yes	100 (15.5)	89 (9.5)	0.576	0.424	0.781	0.001

^§^ ≥ 1.03 ( Male) and ≥ 1.30 (Female). ARV: Antiretroviral. Adjusted for age, gender, ethnicity, hypertension, diabetes mellitus, CD4, HIV RNA Load, TC, LDL, HDL, FPG, alcohol consumption, smoking, liver disease, medication with agents including ARV (PI), b-blocker, anti-hyperglycemic. *aOR* Adjusted odds ratio.

On the other hand being female (OR = 0.550, 95% CI = 0.428- 0.707) and Malay (OR = 0.676, 95% CI = 0.464- 0.985), not taking anti- hyperglycemic agents (OR = 0.603, 95% CI = 0.419- 0.870), higher HDL level (OR = 0.485, 95% CI = 0.384- 0.612), having hepatitis disease (OR = 0.576, 95% CI = 0.424- 0.781) significantly reduce the risk of hypertriglyceridemia (p < 0.001). CD4 cell, viral load, LDL level and smoking were not associated with high TG level significantly (p > 0.05).

### Risk factors of increased LDL

In this study age, gender, taking ARV agents, medication with anti-hyperglycemic drugs, diabetes, smoking, alcohol consumption, CD4 cell, viral load and FPG level were not significant risk factor for high LDL (p > 0.05). Having hypertension (OR = 1.405, 95% CI = 1.093 –1.805) and diabetes mellitus (OR = 1.532, 95% CI = 1.150–2.040), higher TC level (OR = 6.468, 95% CI = 5.319–7.866) with following strong risk as normal HDL level (OR = 2.331, 95% CI = 1.812–2.997) increased the risk (p < 0.05) for high LDL level (Table 3).

**Table 3:** Risk factors for increased low-density lipoprotein cholesterol (LDL-C) level in 1578 HIV subjects (normal = 1023, increased LDL-C = 555) on ARV medication

**Characteristics**	**Normal**	**Increased LDL**	**aOR**	**95.0 C.I. for OR**		**p-value**
	**Mean ± S.D.**	**Mean ± S.D.**		**Lower**	**Upper**	
	**N (%)**	**N (%)**				
**Ethnicity**						
Malay	703 (69.3)	317 (57.4)	1.00			
Chinese	214 (21.1)	175 (31.7)	0.551	0.434	0.701	0.001
Indian	98 (9.7)	60 (10.9)	0.749	0.513	1.093	0.134
**Hypertension**						
No	785 (80.5)	400 (74.6)	1.00			
Yes	190 (19.5)	136 (25.4)	1.405	1.093	1.805	0.008
**TG** (mmol/l)	2.54 ± 2.00	2.14 ± 1.14	0.858	0.800	0.920	0.001
<3.36	413 (40.4)	233 (42.0)	1.00			
≥3.36	609 (59.6)	322 (58.0)	0.937	0.760	1.156	0.545
**TC** (mmol/l)	4.74 ± 1.00	6.26 ± 1.09	6.468	5.319	7.866	0.001
<5.17	705 (69.0)	19 (3.4)				
>5.17	317 (31.0)	536 (96.6)	2.620	2.399	2.861	0.001
**HDL** (mmol/l)	1.31 ± 0.65	1.33 ± 0.33	1.049	0.876	1.257	0.602
Low	349 (34.1)	101 (18.2)	1.00			
Normal^§^	673 (65.9)	454 (81.8)	2.331	1.813	2.997	0.001
**Hepatitis**						
No	882 (86.2)	507 (91.4)	1.00			
Yes	141 (13.8)	48 (8.6)	0.592	0.419	0.836	0.003

^§^ ≥1.03 (Male) and ≥1.30 (Female). Adjusted for age, gender, ethnicity, hypertension, diabetes mellitus, CD4, HIV RNA Load, TC, TG, HDL, FPG, alcohol consumption, smoking, liver disease, medication with agents including ARV (PI), b-blocker, anti-hyperglycemic. *aOR* Adjusted odds ratio.

Conversely being Chinese (OR = 0.551, 95% CI = 0.434–0.701), lower TG level (OR = 0.858, 95% CI = 0.800–0.920), and having hepatitis disease (OR = 0.592, 95% CI = 0.419–0.836), significantly protect the subjects from increased LDL level (p < 0.001).

### Risk factors of increased TC

Also the findings of this study (Table 4) confirmed that that significant risk factors (p < 0.001) for elevated TC were being older (OR = 1.010 95% CI = 1.000–1.021), being female (OR = 1.500, 95% CI = 1.163–1.936), having hypertension (OR = 1.440, 95% CI = 1.122–1.848), having viral load level < 20 copies/mm^3^ (OR = 1.460, 95% CI = 1.093–1.951), higher level of LDL (OR = 6.977, 95% CI = 5.705–8.534), higher TG level (OR = 1.312, 95% CI = 1.220–1.412), having normal HDL level (OR = 1.867, 95% CI = 1.498–2.327) and treatment with PI (OR = 1.561, 95% CI = 1.123–2.169).

**Table 4:** Risk factors for increased total cholesterol (TC) level in 1582 HIV subjects (normal = 725, increased TC = 857) on ARV medication

**Characteristics**	**Normal**	**Increased TC**	**aOR**	**95.0 C.I. for OR**		**p-value**
	**Mean ± S.D.**	**Mean ± S.D.**		**Lower**	**Upper**	
	**N (%)**	**N (%)**				
**Age (year)**						
Mean ± SD	43.87 ± 10.09	44.88 ± 9.65	1.010	1.000	1.021	0.043
**Gender**						
Male	608 (83.9)	665 (77.6)	1.00			
Female	117 (16.1)	192 (22.4)	1.500	1.163	1.936	0.002
**Hypertension**						
No	565 (81.6)	621 (75.5)	1.00			
Yes	127 (18.4)	201 (24.5)	1.440	1.122	1.848	0.004
**ARV medication**						
Without PI	649 (91.3)	731 (87.0)	1.00			
With PI	62 (8.7)	109 (13.0)	1.561	1.123	2.169	0.008
**Viral load (copies/mL)**						
≥20	115 (15.9)	98 (11.4)	1.00			
<20	610 (84.1)	759 (88.6)	1.460	1.093	1.951	0.010
**LDL (mmol/l)**	2.37 ± 0.72	3.57 ± 0.97	6.977	5.705	8.534	0.001
<3.36	705 (97.4)	317 (37.2)	1.00			
≥3.36	19 (2.6)	536 (62.8)	20.150	12.928	31.406	0.001
**TG (mmol/l)**	2.03 ± 1.29	2.73 ± 2.09	1.312	1.220	1.412	0.001
<5.17	363 (50.1)	283 (33.1)	1.00			
≥5.17	361 (49.9)	571 (66.9)	2.029	1.655	2.487	0.001
**HDL (mmol/l)**	1.31 ± 0.70	1.32 ± 0.40	1.034	0.866	1.234	0.714
Low	256 (35.6)	196 (22.9)	1.00			
Normal^§^	466 (64.4)	661 (77.1)	1.867	1.498	2.327	0.001
**Hepatitis**						
No	612 (84.4)	781 (91.1)	1.00			
Yes	113 (15.6)	76 (8.9)	0.527	0.387	0.718	0.001

^§^ ≥1.03 (Male) and ≥1.30 (Female). ARV: Antiretroviral. Adjusted for age, gender, ethnicity, hypertension, diabetes mellitus, CD4, HIV RNA Load, TG, LDL, HDL, FPG, alcohol consumption, smoking, liver disease, medication with agents including ARV (PI), b-blocker, anti-hyperglycemic. *aOR* Adjusted odds ratio.

Interesting that having hepatitis disease (OR = 0.527, 95% CI = 0.387–0.718) significantly protect the subjects from elevated total cholesterol (p < 0.001). Other factors including ethnicity, b-blocker, anti-hyperglycaemic medications, diabetes mellitus, FPG, CD4, smoking and alcohol consumption did not result in high level of TC significantly (p > 0.05).

### Risk factors of low HDL

Also Table 5 shows that the risk of low HDL level was significantly (p < 0.001) related to the factors including being Chinese (OR = 1.753, 95% CI = 1.187–2.589), taking anti-hyperglycemic agent (OR = 1.636, 95% CI = 1.152–2.323), higher LDL level (OR = 1.709, 95% CI = 1.516–1.926), increased level of TC ≥ 5.17 (OR = 1.867, 95% CI = 1.498–2.327) and ARV therapy with PIs (OR = 1.449, 95% CI = 1.037–2.024). Beside this study showed that being Indian (OR = 0.570, 95% CI = 0.386–0.842), higher level of TG (OR = 0. 693, 95% CI = 646–0.745) and having hepatitis disease (OR = 0.630, 95% CI = 0.459–0.865) significantly decreased the risk of low HDL level (p < 0.001). Age, gender, hypertension, b-blocker, diabetes mellitus, FPG, alcohol consumption, smoking were not associated with low level of HDL.

**Table 5:** Risk factors for low high-density lipoprotein (HDL) level in 1582 HIV subjects (normal = 1128, low HDL = 454) on ARV medication

**Characteristics**	**Normal**	**Low HDL**	**aOR**	**95.0 C.I. for OR**		**p-value**
	**Mean ± S.D.**	**Mean ± S.D.**		**Lower**	**Upper**	
	**N (%)**	**N (%)**				
**Ethnicity**						
Malay	105 (23.3)	285 (25.4)	1.00			
Indian	62 (13.7)	96 (8.6)	0.570	0.386	0.842	0.009
Chinese	284 (27.8)	739 (72.2)	1.753	1.187	2.589	0.005
**ARV medication**						
Without PI	382 (86.2)	998 (90.1)	1.00			
With PI	61 (13.8)	110 (9.9)	1.449	1.037	2.024	0.029
**Anti-hyperglycaemic Agents**						
Not taking	57 (12.6)	91 (8.1)	1.00			
Taking	397 (87.4)	1037 (91.9)	1.636	1.152	2.323	0.006
**Viral Load (copies/mL)**						
<20	391 (86.1)	979 (86.8)	1.00			
>20	63 (13.9)	149 (13.2)	0.945	0.688	1.297	0.724
**CD4 (cells/mm3)**						
<200	25 (5.6)	90 (8.0)	1.00			
≥200	419 (94.4)	1029 (92.0)	0.682	0.432	1.078	0.099
**TG (mmol/l)**	2.08 ± 1.37	3.23 ± 2.39	0.693	0.646	0.745	0.001
<5.17	95 (21.1)	551 (48.9)	1.00			
≥5.17	356 (78.9)	576 (51.1)	0.279	0.216	0.360	0.001
**LDL (mmol/l)**	3.17 ± 1.01	2.64 ± 1.05	1.709	1.516	1.926	0.001
<3.36	349 (77.6)	673 (59.7)	1.00			
≥3.36	101 (22.4)	454 (40.3)	2.331	1.813	2.997	0.001
**TC (mmol/l)**						
<5.17	258 (56.8)	466 (41.3)	1.00			
≥5.17	196 (43.2)	661 (58.7)	1.867	1.498	2.327	0.001
**Hepatitis**						
No	383 (84.4)	1010 (89.5)	1.00			
Yes	71 (15.6)	118 (10.5)	0.630	0.459	0.865	0.004

Adjusted for age, gender, ethnicity, hypertension, diabetes mellitus, CD4, HIV RNA Load, TC, TG, LDL, FPG, alcohol consumption, smoking, liver disease, medication with agents including ARV (PI), b-blocker, anti-hyperglycemic. *aOR* Adjusted odds ratio, *ARV* Antiretroviral.

### Risk factors of raised FPG

Table 6 present those significant factors (p < 0.001) which increase the risk of hyperglycemia including increasing age (OR = 1.048, 95% CI = 1.036–1.059), having hypertension (OR = 1.954, 95% CI = 1.522–2.508) and higher level of TG (OR = 1.130, 95% CI = 1.066–1.199).

**Table 6:** Risk factors for increased fasting plasma glucose (FPG) level in 1540 HIV subjects (normal = 952, increased FPG = 588) on ARV medication

**Characteristics**	**Normal**	**Hypertensive**	**aOR**	**95.0 C.I. for OR**		**p-value**
	**Mean ± S.D.**	**Mean ± S.D.**		**Lower**	**Upper**	
	**N (%)**	**N (%)**				
**Age (year)**						
Mean ± SD	42.79 ± 9.68	47.17 ± 9.56	1.048	1.036	1.059	0.001
**Gender**						
Male	751 (78.9)	494 (84.0)	1.00			
Female	201 (21.1)	94 (16.0)	0.711	0.543	0.931	0.013
**Hypertension**						
No	754 (82.6)	398 (70.8)	1.00			
Yes	159 (17.4)	164 (29.2)	1.954	1.522	2.508	0.001
**b-Blocker**						
taking	43 (4.5)	44 (7.5)	1.00			
Not Taking	909 (95.5)	544 (92.5)	0.585	0.379	0.902	0.014
**Anti-hyperglycaemic**						
Taking	35 (3.7)	111 (18.9)	1.00			
Not taking	917 (96.3)	477 (81.1)	0.164	0.110	0.244	0.001
**Diabetes mellitus**						
Yes	96 (10.1)	149 (25.3)	1.00			
No	856 (89.9)	439 (74.7)	0.330	0.249	0.438	0.001
**TG (mmol/l)**	2.25 ± 1.74	2.65 ± 1.82	1.130	1.066	1.199	0.001
<5.17	432 (45.4)	197 (33.6)	1.00			
≥5.17	519 (54.6)	389 (66.4)	1.644	1.327	2.035	0.001
**Hepatitis**						
No	857 (90.0)	498 (84.7)	1.00			
Yes	95 (10.0)	90 (15.3)	1.630	1.197	2.220	0.002

Adjusted for age, gender, ethnicity, hypertension, diabetes mellitus, CD4, HIV RNA Load, TC, TG, LDL, HDL, alcohol consumption, smoking, liver disease, medication with agents including ARV (PI), b-blocker, anti-hyperglycemic. *aOR* Adjusted odds ratio.

On the other hand being female (OR = 0.711, 95% CI = 0.543- 0.931), not taking b-blocker (OR = 0.585, 95% CI = 0.379- 0.902), not taking anti-hyperglycemic agents (OR = 0.164, 95% CI = 0.110–0.244) and not having diabetes mellitus (OR = 0.330, 95% CI = 0.249–0.438) significantly protect the subjects from increased fasting plasma glucose (p < 0.001).

Differences in ethnicity, using PI, CD4 viral load level, LDL, TC, HDL, alcohol consumption and smoking were not increased the risk of high FPG significantly (p > 0.05).

## Discussion

This primarily and large study of HIV infected subject on ART found that dyslipidemia was a high predominant disorder. Majority of the subjects (60%) had increased levels of TG or TC while a less proportions of subject affected with high levels of LDL (35.1%) and FPG (38.2%) and, low HDL level (28.7%). Some studies in Thailand reported a similar rate of dyslipidemia at 53.6% and 88% [38,50]. Other studies from other developing countries showed similar results oh high prevalence lipid abnormalities rate of more than 76% [35] from Tanzania, 82.3% [19] from Southern Ethiopia, 20-100% from India [7,41].

Disturbances in level of triglyceride and total cholesterol were more prevalent than HDL and LDL in this study. Generally increases in TG and TC level are mainly attributed to treatment with PIs. The multiple mechanism of PIs in developing lipid and glucose abnormalities include reduction in catabolism and increase in production of very low density lipoprotein [51,52], impaired catabolism of fatty free acid [53], increased synthesis of triglyceride in liver [54], decreased expression of LDL receptors [55], interference with the intercellular process regulating glucose and lipid metabolism in insulin-response tissue [56]. Other relevant factors comprise of aging, abdominal obesity, diabetes, lifestyle, gender and ethnic differences, type and length of time on ARV regimen can result in various incidence/prevalence of lipid and glucose abnormalities.

Aging, race and gender differences are well-known irreversible risk factors for metabolic abnormalities. Elderly population has lipid and glucose metabolism changes due to liver cell dysfunctions [57]. The beneficial effect of endogenous estrogen women before menopause causes less lipid abnormalities [58]. Similar to this study Richter and colleagues [59] in a cohort study indicated that age, treatment with PI and male gender were risks for dyslipidemia. Some studies [60,61] which were conducted in the United State also revealed that race/ethnicity was a highly significant predictor of plasma lipids. In a study the increased lipid level was significantly less associated with Malay and Chinese’s race [62]. One of the possible explanations may be due to the lower prevalence of abdominal obesity among Malay and Chinese HIV patients [62]. In the present study alcohol consumption was associated with high level of TG. As the recognized dietary risk for high TG, alcohol is rich in calorie and disturb the liver function, the human organ which contributes in metabolize of the nutrients [63], thus it can be stored in body as fat and increase in blood as TG. It is important to stress that the data are derived from a male population mostly and that their relevance is mainly to be referred to male sex.

Interestingly the effect of some risk or protective factors on lipid and glucose can be explained by their indirect and intermediary role. In this study higher CD4 cell count was a risk factor for high TG while viral load level < 20 (copies/mm^3^) raised the risk of high TC. ARV medications boost immune system by increasing in CD4 cells and diminishing HIV vial load thus the effect of ARV medication on lipids was seen through the raised CD4 and decreased viral load [64]. We also found that treatment with anti-hyperglycemic agents was a significant associated risk for high TG and low HDL level. Since diabetic patients have more lipid abnormalities thus taking anti-hyperglycaemic medication can make a connection between diabetes and these lipid disorders indirectly [65]. Approximately 14% of our population had hepatitis as liver diseases who had lowered risk of lipid disorders and increased possibility of high FPG. On the hepatitis virus nature, some studies found that hepatitis viral replication during its metabolic processes can drop in lipid levels by interrupting cholesterol synthesis and using host lipids for replication, decreasing circulating lipids, and clearance of the virus results in rebound increase of lipid levels [66,67]. Since liver is the main body organ for balancing plasma glucose, damage to the liver cell by hepatitis virus causes disturbed liver function and insulin production [68] thus it is expected the elevated FPG during hepatitis infection.

### Limitations of the study

Comprehensive blood lipid and glucose evaluation were not assessed in this study just for those with current tests (year 2012). Also inability of assessment of anthropometrics including waist circumference, body mass index (BMI) and body fat percentage, the cross-sectional nature of the study, small number of female as well Indian subjects, and lack of ARVnaïve or HIV negative subjects as controls, absence of previous published studies in lipid abnormalities among Malaysian population with HIV/AIDS in order to comparison with the present study were considered as other limitations in this study.

## Conclusion

Our study indicates a high proportion of HIV-infected patients receiving ARV medication met the criteria for dyslipidemia. Also treatment with protease inhibitorswas responsible and risk factor for high prevalence of lipid profile disorders. Therefore, it is crucial to evaluate and monitor these abnormalities before initiation and during Highly Active Antiretroviral therapy (HAART) to monitor any rising trends. It should be mentioned that study of metabolic abnormalities among ARV naive patients for assessment of independent effect of HIV itself on lipid and glucose is considerable due to the importance of the choice of type ARV agents in combination therapy as HAART.Moreover based on our results; assessment of long-term effects of ARVagents on lipid abnormalities is suggested. Also investigations and implementation programs on prevention and treatment of lipid abnormalities by the way of lifestyle and nutritional modification strategies are optional.

## Abbreviations

AIDS: Acquired immune deficiency syndrome; ART: Antiretroviral therapy; ARV: Antiretroviral; BMI: Body mass index; FPG: Fasting plasma glucose; HAART: Highly active antiretroviral therapy; HDL: High-density lipoprotein; HIV: Human immunodeficiency virus; LDL: Low-density lipoprotein; TC: Total cholesterol; TG: Triglyceride.

## Competing interests

The authors declared that they have no competing interest.

## Authors’ contributions

HN and RR designed research; HN conducted research and analysed data; KCLC supervised hospital settings. All authors read and approved the final manuscript.

## Pre-publication history

The pre-publication history for this paper can be accessed here:

http://www.biomedcentral.com/1471-2458/13/758/prepub
